# Multi-Platform Software for Electrical and Microstructural Analysis of Silicon Solar Cell Metallization

**DOI:** 10.3390/ma19132717

**Published:** 2026-06-24

**Authors:** Małgorzata Musztyfaga-Staszuk, Dušan Pudiš, Rafał Honysz

**Affiliations:** 1 Materials Investigating Laboratory, Faculty of Mechanical Technology, Silesian University of Technology, Konarskiego 18A Street, 44-100 Gliwice, Poland; 2 Department of Physics, Faculty of Electrical Engineering and Information Technology, University of Žilina, Univerzitná 8215/1, 010 26 Zilina, Slovakia; dusan.pudis@uniza.sk; 3 Department of Engineering Materials and Biomaterials, Faculty of Mechanical Technology, Silesian University of Technology, Konarskiego 18A Street, 44-100 Gliwice, Poland; rafal.honysz@polsl.pl

**Keywords:** silicon solar cells, metallization, microstructural characterization, confocal microscopy, electrical resistivity, educational software, virtual laboratory

## Abstract

This paper presents proprietary, multi-platform software developed in Python for analyzing the electrical and microstructural properties of silicon solar cell metallization. Utilizing a sample set of 20 commercial solar cells, electrical resistivity and contact resistance measurements obtained via the potential difference method were correlated with high-resolution topographic data from AFM, SEM, and CLSM. This process enabled the quantification of how specific features, such as surface roughness and finger height, directly influence electrical performance. The developed algorithms offer high-fidelity predictive capabilities, with relative errors below 4%. This “virtual laboratory” serves as a transformative research and educational tool, allowing for complex materials analysis while avoiding the necessity for destructive testing.

## 1. Introduction

In the last five years, the photovoltaic industry has transitioned from simplified one-dimensional (1D) analysis to complex multi-dimensional simulations to bridge the gap between ideal laboratory conditions and mass production [1,2]. The release and subsequent updates of Quokka3 have redefined system-level modelling by providing a comprehensive device physics approach that accounts for 3D boundary effects, which are often neglected in traditional frameworks [3,4]. However, recent validation studies indicate that discrepancies still exist when comparing results with experimental data for advanced bifacial architectures [3,4]. While Sentaurus TCAD remains the gold standard for high-fidelity physical modelling, standard TCAD often fails to predict “*local shunting*” because it assumes an idealised, perfectly smooth interface between the silver paste and the silicon emitter [5,6]. Consequently, identifying inhomogeneous contact resistance has become a critical research priority, as it represents a significant bottleneck in PERC and TOPCon efficiency [5,6,7].

The calculation of specific electrical properties, such as resistance and resistivity at the metal–semiconductor junction, requires moving beyond treating contact resistance as a static parameter [7,8]. Modern predictive modelling must incorporate variables influenced by the firing temperature, silver paste chemistry, and the resulting structural layout, including a “*reaction-layer*” component to ensure accuracy [7,8,9,10]. Furthermore, the physical shape of front fingers produced via screen printing is rarely a perfect rectangle, and these inhomogeneous finger shapes lead to non-uniform current density [9,10,11,12]. This is particularly problematic in TOPCon cells, where 2D approximations fail to capture the impact of finger interruptions, potentially leading to significant errors in predicted efficiency [11,12].

Beyond electrical properties, the trade-off between conductivity and optical transparency remains a primary focus of recent literature [12]. As industry moves toward narrower fingers and higher busbar counts in MBB (Multi-Busbar) cells, the complexity of shading simulations increases, requiring much more rigorous computation to avoid overestimating optical gains [13]. Older tools, such as PC1D, are fundamentally limited in capturing edge recombination and grid shading, necessitating a transition to more robust 3D modelling [14,15,16]. Future software must therefore be flexible enough to simulate non-standard grid patterns and innovative metallisation geometries beyond the traditional H-pattern [17,18].

The current project addresses these challenges by developing custom software in Python, mirroring a wider academic trend toward open-source, modular simulation environments [17,18,19]. Python’s ability to handle large datasets makes it ideal for integrating structural data-such as that obtained from Scanning Electron Microscopy (SEM) directly into electrical solvers to calculate local resistivity based on measured contact areas [19,20]. Furthermore, this modular approach allows software to be scaled for educational purposes, providing rigorous preliminary research tools for students, as demonstrated by the Talent Detector initiative [9]. Ultimately, the modelling of front metallisation shifts from “*device-level averages*” to “*interface-level specifics*”. The development of a dedicated Python-based tool for calculating resistance and optical properties is therefore highly aligned with current scientific trends and the industrial need to accurately represent screen-printing inhomogeneities while avoiding critical 1D/2D approximation errors.

## 2. Research Material

**Figure 1:**
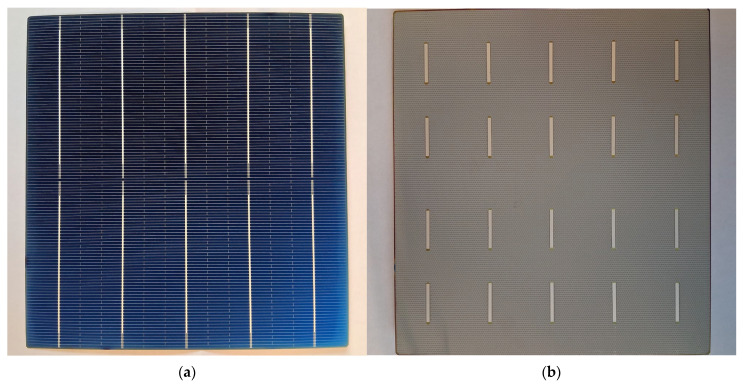
Visual representation of the examined multicrystalline silicon solar cells with five busbars (5BB): (**a**) front side and (**b**) rear side of the solar cell.

Twenty commercial multicrystalline silicon solar cells with five busbars (5BB) were used for the research. These cells featured an area of 15.8 cm × 15.8 cm ± 0.25 mm and a thickness of 180 ± 30 μm (Figure 1).

**Figure 2:**
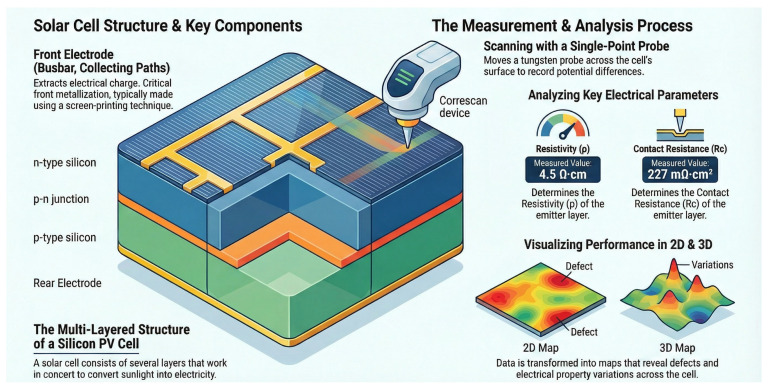
Anatomy of a solar cell: Measuring electrical performance.

Generative AI tools were utilized in the preparation of this study: Napkin AI (web version, accessed May 2026) was employed to generate the illustrative diagram in Figure 2, and OpenAI (GPT-5.5, accessed May 2026) was used for language checking and grammatical verification of the manuscript.

## 3. Methodology

### 3.1. Structural and Chemical Analysis

To assess the surface topography and cross-sectional morphology of the front electrode and silicon substrate, scanning electron microscopy (SEM) was utilized. This analysis revealed a textured silicon surface with pyramids reaching an average height of approximately 3 µm. Furthermore, energy-dispersive X-ray spectroscopy (EDS) was applied to determine the chemical composition of specific micro-areas, such as the busbars and substrate. Optical properties, such as reflection, were measured using a UV-VIS-NIR Lambda 950S spectrophotometer (PerkinElmer Inc., Waltham, MA, USA). The surface topography of the selected sample was measured using an atomic force microscope (AFM) Park Systems XE-100 (Park Systems Corp., Suwon, Gyeonggi, Republic of Korea) in non-contact mode. The silicon surface and the top contact surface were scanned. For confocal laser scanning microscope (CLSM) analysis, a Keyence VK-X200 (Keyence Corporation, Osaka, Japan) Series system with a 405 nm laser and 50× and 150× objectives were used.

### 3.2. Electrical Characterization via Correscan

Figure 2 is a diagram illustrating the cell structure and the process of measuring electrical parameters. Figure 2 was prepared using the generative artificial intelligence tool Napkin AI (Napkin, Inc.). The tool was used exclusively to generate the graphical layout and visual representation of concepts based on author-provided instructions. All scientific content, terminology, relationships between concepts, and final figure designs were re-viewed, verified, and approved by the authors. The authors take full responsibility for the accuracy and integrity of the figure and confirm that the use of the AI tool did not affect the research results, data analysis, or scientific conclusions presented in this study.

Local electrical properties were mapped using the Corescan device (manufactured by Mechatronics/SunLab) in “Core Scan” (Contact Resistance Scanner) mode. This method utilizes potential difference (PD) measurements to evaluate the quality of the metal–semiconductor junction. The experimental station is equipped with the CoreScan device, designed by SunLab (Petten, the Netherlands) and manufactured by R&R Mechatronics (Zwaag, the Netherlands) under a SunLab license.

During the procedure, the illuminated solar cell is short-circuited by a 1 Ω resistor, and a tungsten single-point probe (0.2 mm diameter) scans the surface along a predefined trajectory. The probe must penetrate non-conductive layers (ARC and passivation) to establish direct contact with the emitter.

**Figure 3:**
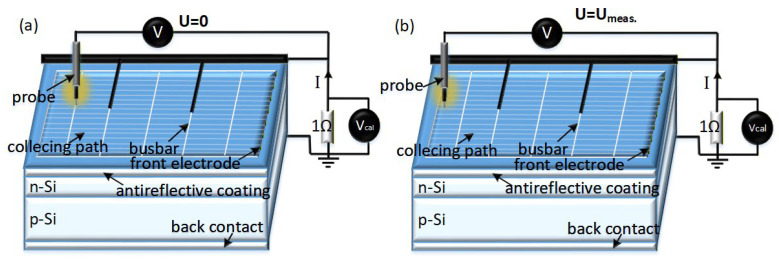
Experimental arrangement for monitoring variations in emitter-layer contact resistance and resistivity; a scanning measurement probe contacting: (**a**) the current-collecting track of the top electrode, (**b**) the emitter surface in the region between the tracks.

The measurement procedure utilizing the Corescan device (which is part of the measurement setup equipment) relies on recording variations in potential as a moving tungsten probe follows a predefined trajectory across the front surface of the solar cell. During this process, the probe identifies three distinct electrical states based on its contact point (Figure 3):

- *Contact with the collecting path:* When the probe is in contact with a collecting finger or track, it remains at the same potential as the busbar, resulting in a recorded potential difference of Δ*U* = 0.
- *Contact with the metal path–emitter transition region:* When the probe touches the interface between the metal electrode and the semiconductor, the recorded potential difference increases sharply. This rapid rise (Δ*U* > 0) is a diagnostic indicator of a properly executed co-firing and fusion operation for the conductive path.
- *Contact with the emitter surface*: When the probe moves onto the emitter surface of the cell, a characteristic local potential difference is recorded, expressed by the condition Δ*U* > *U_meas_*.

This technique is considered destructive, as the probe must pierce the non-conductive antireflective (ARC) and passivation layers to establish the direct contact required to map these voltage drops across the illuminated cell.

**Figure 4:**
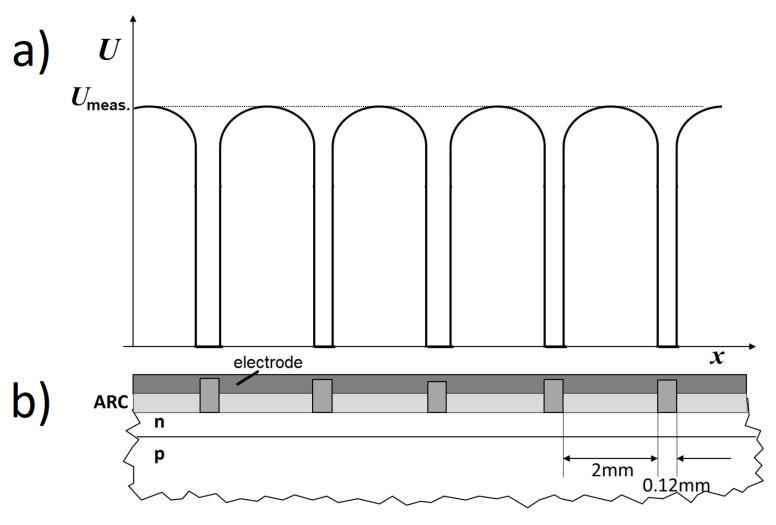
Potential response (**a**) recorded during tungsten probe scanning of a photovoltaic cell surface (**b**). Contact with the electrode collecting path results in U = 0, while contact with the emitter surface produces a potential of U = U_meas_.

Figure 4 presents the potential changes versus the tungsten probe position on the photovoltaic cell surface.

Key parameters were calculated using a scanning current density of 30 mA/cm^2^ and a correction factor (C) of 1.8 to account for shading and current leakage:

Resistivity (ρ) of the transition region between the metal electrode path and the emitter:

(1) $$\rho =C\cdot \frac{U_{ce}}{d\cdot J_{sc}},\;\Omega \cdot cm$$

where $U_{ce}$ is the recorded potential, d is the distance between the collecting paths of the solar cell probe, and $J_{sc}$ is the scanning contact current density.

Actual Contact Resistance (R_c_):

(2) $$R_{c}=C\cdot \frac{U_{ce}\cdot w}{J_{ce}\cdot d},\Omega \cdot cm^{2}$$

where w—represents the path width.

### 3.3. Computational Support and Visualization

The development of virtual tools that simulate research equipment and methodologies provides a foundation for integrating laboratory research, simulation, measurement, and education. Utilizing these tools facilitates the transfer of research and teaching procedures from physical laboratories to virtual environments. Access to free, publicly available software encourages user engagement, thereby increasing the number of experiments conducted virtually and enhancing the overall effectiveness of such research [18,19].

Data processing and visualization were handled by proprietary software developed in Python (version 3.12). The application utilizes the Kivy and KivyMD frameworks to provide an intuitive interface for simulating the manufacturing process and generating 2D maps and 3D images of the potential distribution. This visualization allows for the identification of technological defects, such as non-uniform doping or high-resistance areas (indicated by potential > 25 mV).

Measurement error analysis is an essential component for verifying the precision of the calculated electrical parameters of photovoltaic cells. To assess the quality and accuracy of the measurements, the developed software utilizes the following relationships for absolute and relative errors.

1. Absolute Error (Δx)

The absolute error is defined as the difference between the measured (or approximate) value and the true (exact) value. It allows for determining the physical magnitude of the measurement’s deviation from the reference.

Formula:

(3) $$\Delta x=|x_{\mathrm{measured}}-x_{\mathrm{true}}|$$

where $x_{\mathrm{measured}}$ is the value recorded during the measurement, $x_{\mathrm{true}}$ is the true or exact value.

2. Relative Error (δ)

The relative error is the ratio of the absolute error to the true (or approximate) value. This parameter is critical for evaluating the quality of the measurement, as it shows the scale of the error in relation to the magnitude of the measured quantity. It is usually expressed as a percentage.

Formula:

(4) $$\delta [\%]=\frac{\Delta x\cdot 100\%}{x_{\mathrm{true}}}\;$$

where Δx is the absolute error.

3. Application in Research

These error calculations are integrated into the proprietary software developed in Python. Once the measured and calculated values are entered into the designated fields of the “Error Calculator” section, the system automatically computes both error types. This functionality enables real-time diagnostics of the accuracy of the measuring apparatus and the correctness of the cell metallization process. The results are archived within the system’s history, allowing for subsequent statistical analysis of the measurement series.

## 4. Results and Discussion

### 4.1. SEM and EDS Analysis of the Solar Cells

**Figure 5:**
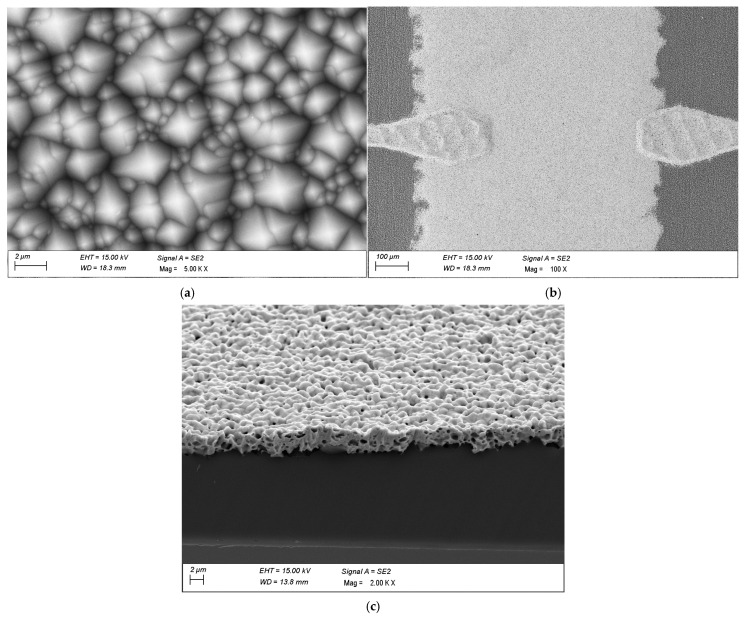
Microstructural SEM analysis of a silicon solar cell: (**a**) surface topography of the textured silicon substrate, (**b**) surface topography of the silver front electrode, and (**c**) cross-sectional view of the contact region between the electrode and the silicon substrate.

Microstructural analysis using scanning electron microscopy (SEM) allowed for a comprehensive evaluation of the surface morphology and the contact region of the investigated solar cells (Figure 5).

Topographic studies of the silicon substrate revealed the presence of a regular pyramidal texture with an average pyramid height ranging from 2.5 to 3 μm (Figure 5a). Regarding the front metallization, SEM observations (Figure 5b) revealed that the electrodes made from commercial silver paste are characterized by a distinctly porous surface structure. Despite this porosity, fractographic analysis of the cross-sections (Figure 5c) confirmed the formation of a continuous contact between the silver electrode and the silicon substrate. This interface quality is a necessary condition for achieving low series resistance and high charge collection efficiency (Figure 5c).

**Figure 6:**
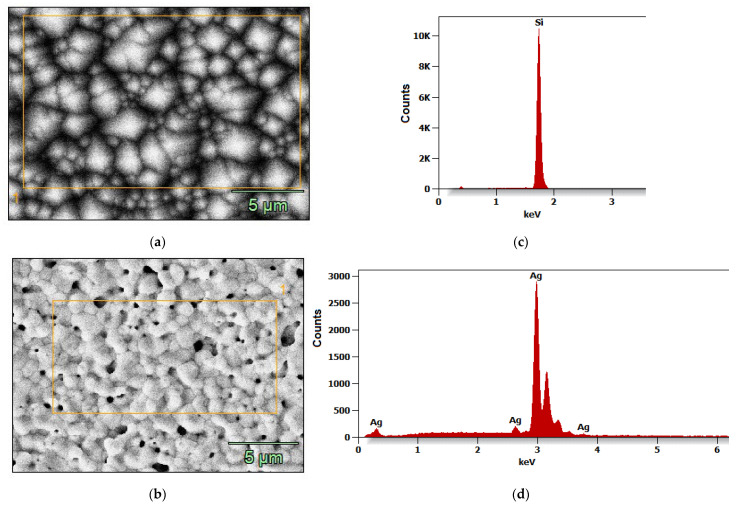
Results of energy-dispersive X-ray spectroscopy (EDS) for selected micro-areas of the solar cell, including spectra from: (**a**) the surface; (**b**) the busbar; (**c**) [Si]; (**d**) [Ag].

EDS analysis of selected areas of the 5BB solar cell is reported in Table 1 and Figure 6.

### 4.2. Electrical Measurements of the Solar Cells

#### 4.2.1. Analysis of I–V Characteristics

**Figure 7:**
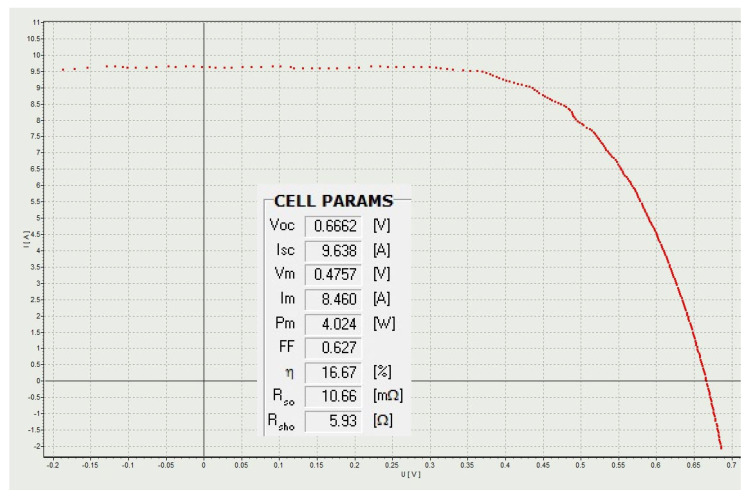
Current–voltage (I–V) characteristic of a high-efficiency 5-busbar (5BB) solar cell (Sample No. 4 from Table 2).

As shown in Figure 7, the current–voltage (I–V) characteristic of the high-efficiency 5-busbar (5BB) solar cell (Sample No. 4 from Table 2) demonstrates favorable electrical performance. Table 2 contains the calculated values of electrical parameters from additional software installed on the computer included in the I–V measurement station.

Analysis of Electrical Parameters Based on the data collected in Table 2, significant variations in cell performance were observed, which are directly linked to the metallization structure and series resistance (R_s_).

- *Conversion Efficiency (E_ff_):* The efficiency of the analyzed cells ranged from 15.21% to 16.67%. The highest value (16.67%) was recorded for solar cell no. 4, which also exhibited a high fill factor (FF = 0.627).
- *Series Resistance (R_s_):* A critical factor influencing efficiency was the series resistance measured at U_oc_. These values fluctuated between 10.7 mΩ and 17.3 mΩ. A clear correlation was found cells with the lowest R_s_ values demonstrated the highest maximum power (P_m_) and efficiency.
- *Current–Voltage Parameters:* The open-circuit voltage (U_oc_) for the samples ranged from approximately 662 mV to 676 mV, while the short-circuit current (I_sc_) varied between 9383 mA and 9710 mA.

Impact of Busbar Configuration The results highlight a direct correlation between the front-side metallization design and the electrical output:

1. *Efficiency Trend:* Solar cells with a higher number of busbars show significantly higher maximum power output and greater conversion efficiency. Specifically, the 5BB configuration proved to be the most efficient, while the 1BB configuration (example I–V curve shown in Figure 7) resulted in the lowest efficiency.
2. *Ohmic Loss Reduction:* The data in Table 2 shows that series resistance values decrease as the number of busbars increases. Adding more bus bars reduces the distance charges that must travel through the fingers, thereby lowering the overall resistance of the solar cell.

This initial characterization establishes the fundamental performance metrics of the solar cells. The following sections will provide a deeper insight into the electrical properties by utilizing different measurement devices to examine the microstructural and regional characteristics of the cells.

#### 4.2.2. Determination of Contact Resistance and Resistivity of the Emitter Layer

**Figure 8:**
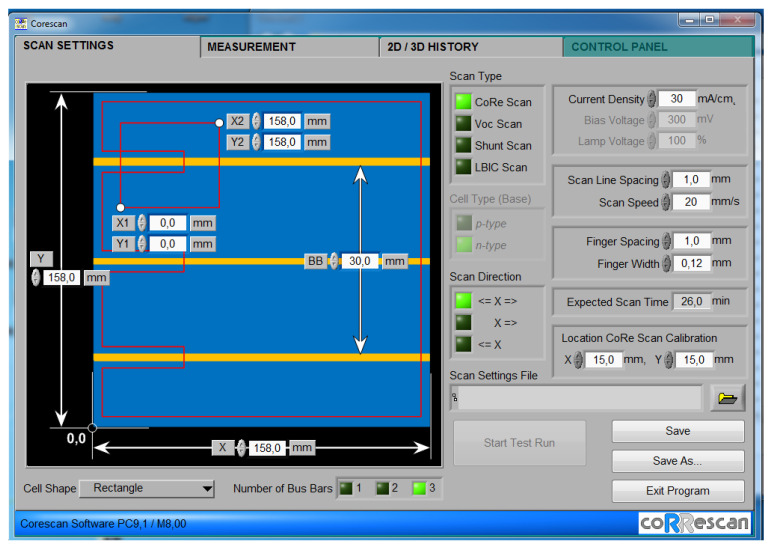
Overview of the software configuration with the Core Scan mode selected before the measurement.

Figure 8 illustrates the computer screen view with input data specifying the measurement parameters of the selected sample solar cell. 2D maps and 3D images provide a detailed view of parameter variations within a photovoltaic cell, which can inform conclusions about the manufacturing process of its components.

**Figure 9:**
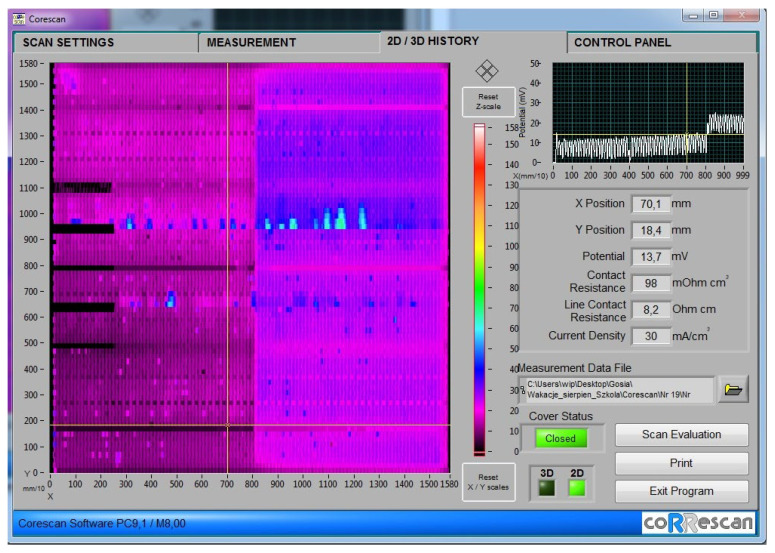
2D Measurement tab displaying the results after the measurement process.

**Figure 10:**
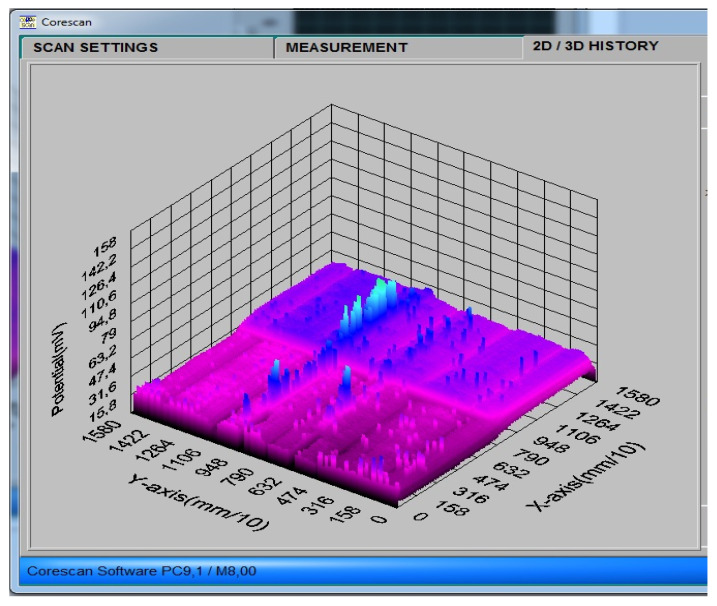
Overview of the 3D History tab.

Representative measurement results are presented in Figure 9 and Figure 10.

To gain a more granular understanding of the resistive loss mechanisms, a comprehensive mapping of all 20 multicrystalline silicon cells (15.8 cm × 15.8 cm) in the 5-busbar (5BB) series was conducted using the Potential Difference (PD) method. This technique reveals spatial non-uniformities at the metal–semiconductor interface that are typically averaged out in standard global current–voltage (I–V) measurements.

**Figure 11:**
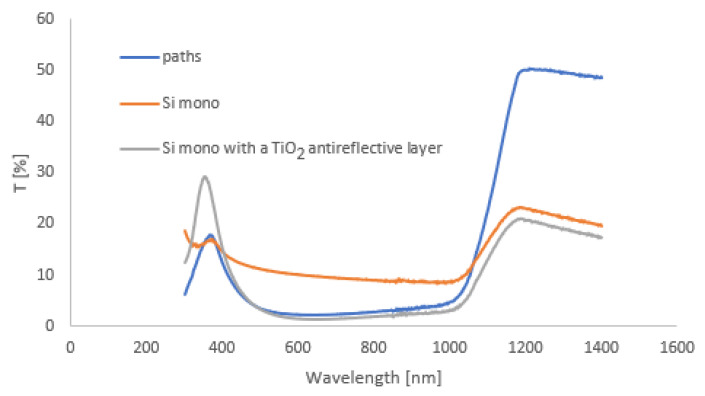
Transmission spectra of a multicrystalline solar cell without coating and with an antireflective layer, measured in the wavelength range of 0–1500 nm using a Lambda 950S spectrophotometer.

**Figure 12:**
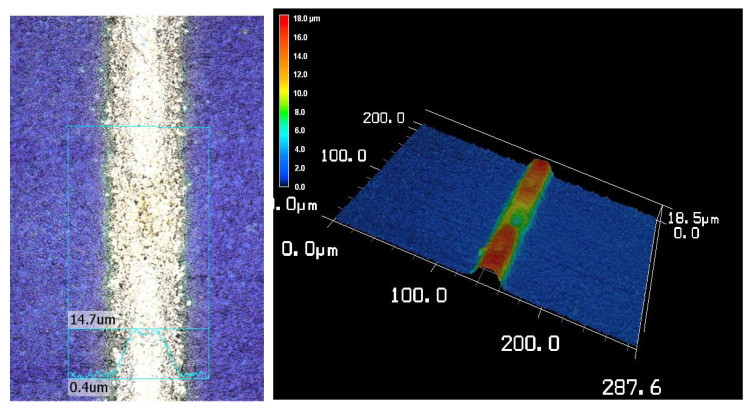

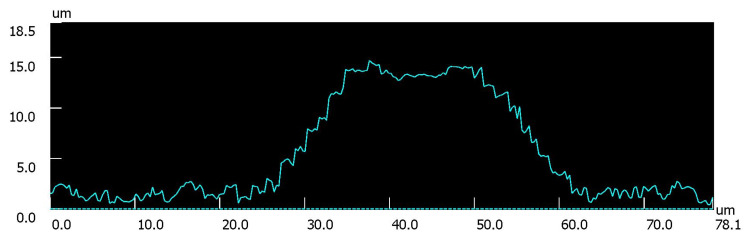
Two- and three-dimensional topography and the average height profile of a busbar (front electrode) made of a commercial material on a silicon substrate (CLSM).

The characterization focused on cells with an n-type emitter featuring a sheet resistance of approximately 50 Ω/□. Measurements were performed with a constant scanning current density (J_sc_) of 30 mA/cm^2^ and a correction factor (C) of 1.8. While potential distribution patterns varied across the entire experimental batch, Figure 10, Figure 11 and Figure 12 illustrate the software configuration and mapping results for a single representative specimen (solar cell no. 4), chosen for its peak conversion efficiency of 16.67%. These variations in local resistance across the series are likely a consequence of technological factors, including screen-printing precision and the temperature distribution during metallization (T_M_), both of which are critical to the final ohmic interface quality.

Data analysis for the 5BB configuration revealed that the resistivity of the transition region (ρ) spanned from 13.5 to 33.4 Ω·cm, while emitter layer contact resistance (R_c_) values were recorded between 162 and 401 mΩ·cm^2^. Additionally, the potential along the collecting tracks (Uce) fluctuated between 22.5 and 55.7 mV.

The 2D and 3D visualizations for the representative cell (Figure 9 and Figure 10) identify specific regions where the potential exceeded a 25 mV threshold, appearing as bright “*hot spots*”. Such anomalies indicate local structural defects potentially stemming from:

1. *Sub-optimal Metallization Temperature:* Improper firing profiles can lead to incomplete glass frit penetration or, conversely, excessive diffusion of paste components, both of which degrade the junction performance.
2. Emitter Doping Fluctuations: Spatial variations in donor concentration across the silicon wafer surface significantly impact the resulting contact uniformity and R_c_ values. Despite these observed local non-uniformities, the optimization of the 5BB metallization design enabled the best-performing units to achieve a low total series resistance (R_s_) of 10.7 mΩ, confirming the high charge collection efficiency provided by increased busbar counts.

#### 4.2.3. Determination of Optical Chosen Parameter

The optical properties of the fabricated structures were evaluated to assess the impact of surface modification on the device’s spectral parameters. As illustrated in Figure 11, the deposition of a dielectric coating results in a clear increase in transmission across the entire measured range, with the most significant changes recorded in the near-infrared (NIR) region (above 1000 nm). In this interval, the transmittance for the sample with the antireflective coating reaches values near 50%, demonstrating the high efficacy of the layer in minimizing optical losses stemming from front-surface reflection.

This spectral characteristic confirms that the application of a properly selected anti-reflective coating (ARC) promotes optimized light trapping within the structure. The reduction in the reflectance coefficient allows a greater number of photons to penetrate the active silicon layer, which is a critical condition for increasing the generated photocurrent density in the device. These results correlate with literature data suggesting the necessity of utilizing materials with a low refractive index to approach the theoretical efficiency limits of modern crystalline silicon (c-Si) solar cells.

### 4.3. Surface Topography Characterization by AFM and CLSM

Figure 12 illustrates the surface morphology of the front electrode. Specifically, the 2D and 3D topography, along with the average height profile of the busbar, were characterized using CLSM to evaluate the deposition quality on the silicon substrate.

**Figure 13:**
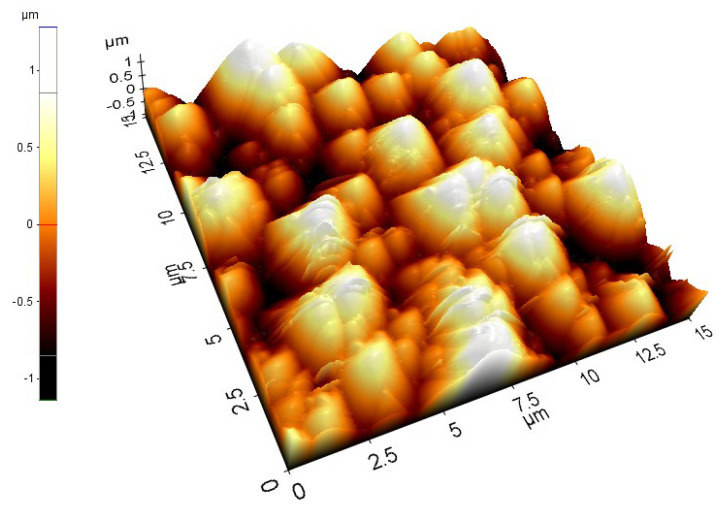
Topography of the textured silicon surface (AFM).

The topography of the textured multicrystalline silicon surface is shown in Figure 13.

AFM analysis confirms that the crystals reach heights of up to 1.8 µm, with comparable lateral dimensions, reflecting the observed crystalline roughness.

**Figure 14:**
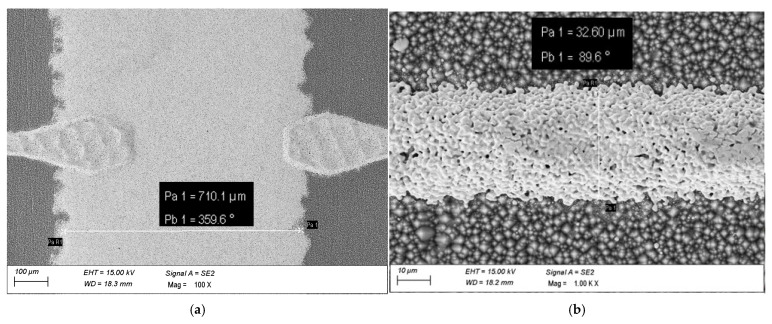
SEM images of the surface topography of the front-side electrode: (**a**) section of the busbar with a collecting path, (**b**) section of the busbar (chosen example).

**Figure 15:**
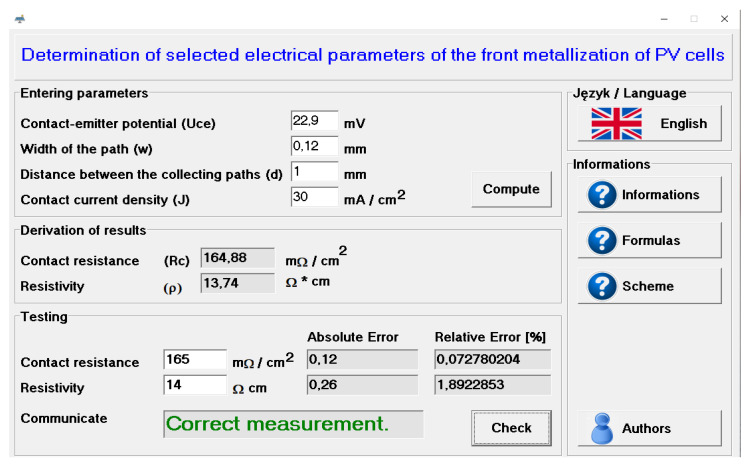
Main application window.

A comparative analysis of Figure 14 and Figure 10 indicates that the dimensions of the front metallization determined through macroscopic methods differ significantly from the results obtained using precise analytical equipment. In Figure 10 (as well as the software configuration shown in Figure 15), the collecting path width was initially entered based on macroscopic observation and nominal settings at 0.12 mm (120 μm). However, the much more accurate microstructural analysis provided by Confocal Laser Scanning Microscopy (CLSM) in Figure 14 reveals the actual dimensions:

- Contact Height: The height is approximately 15 μm (specifically 14.7 μm, as shown on the profile label).
- Path Width: The actual height profile in Figure 14 demonstrates that the path is significantly narrower than macroscopically assumed, measuring approximately 35 μm (inferred from the *X*-axis of the profile, which has a total scanning width of 78.1 μm).

This substantial discrepancy between the nominal value (120 μm) and the actual measured value (~35 μm) confirms that CLSM analysis is essential to avoid errors stemming from 2D approximations. Such precision is critical for the accurate determination of electrical parameters, particularly contact resistance, where the actual contact area directly influences the final calculations.

The CLSM analysis revealed a contact height of approximately 15 µm.

### 4.4. SEM Supplementary Investigations

The SEM investigations were initially performed on the busbar, and based on these observations, the analyzed area and measurement parameters were selected experimentally. Only at a later stage, after additional analyses had been carried out, was the actual width of the collecting path precisely determined to be approximately 33 μm. This value is very close to the result obtained using the CLSM method (~35 μm), which confirms the validity of the performed analyses and the high consistency between both measurement techniques. This is also confirmed by the SEM results presented in Figure 14.

### 4.5. Proprietary Software

The interface is divided into two main tools, the first of which allows the user to calculate contact resistance and resistivity values based on input data, including contact-emitter potential, path width, distance between collecting paths, and contact current density. The minimum and maximum values of these parameters are summarized in Table 3. After entering the correct data and pressing the “*Computed results are displayed*” computed results are displayed. The second tool allows verification of test values or obtained from real measurements. These measurements are compared with the calculated values. and the user receives information about the difference between them in the form of absolute and relative errors.

Tests were conducted to verify the correct operation of the software developed. For this purpose, four samples were selected and twenty measurements were performed on real equipment. The supplier who provided the cells for verification purposes manufactured them all using the same technology. Therefore, the path width is 0.12 mm, the distance between the collecting paths is 1 mm, and the contact current density is 30 mA/cm^2^. This data was entered into the program. Consequently, the only input parameter that changed during the program’s use and cell testing was the contact-emitter potential, which was measured at five different values for each cell. The results are presented in Table 4. The first section, “*Input values*,” contains the cell values on which the measurements were performed. These same values were used as input parameters for the calculations. The second section, “Calculated Values,” presents the results obtained from the software calculations. Contact resistance and resistivity were calculated based on the input values. The “*Measured values*” section contains the actual values recorded during the experiment. The final section compares the obtained results, with absolute and relative error values computed between the calculated and measured values. In all cases, accurate results were obtained for both measurements and calculations. The relative error values did not exceed 4%, which is considered an excellent outcome.

## 5. Conclusions

An integral part of the model validation involved SEM investigations, which revealed a textured silicon surface with pyramids reaching an average height of approximately 2.5–3 μm and a porous, yet continuous, microstructural contact between the silver paste and the substrate. Electrical measurements confirmed the high quality of the analyzed 5BB cells, with conversion efficiencies ranging from 15.21% to 16.67% and a minimum series resistance reaching 10.7 mΩ. The key element for verifying the software’s algorithms was the correlation of electrical data with high-resolution CLSM analysis, which demonstrated that the actual dimensions of the collecting path (width of approx. 35 μm and height of approx. 15 μm) differ significantly from macroscopic assumptions and nominal settings of 0.12 mm. Incorporating these precise microstructural dimensions into the computational model allowed for the reliable determination of local contact resistance, which for the tested series ranged between 162 and 401 mΩ·cm^2^. By integrating microstructural and electrical results, this “virtual laboratory” avoids errors stemming from 2D approximations and serves as a highly accurate research and educational tool.

The results obtained through the developed software and methodology are highly consistent with recent advancements in advanced thin-film deposition and metallization technologies [20,21], providing a robust framework for future collaborative efforts in PV interface optimization.

The findings regarding the impact of metallization architecture on solar cell efficiency and resistive losses are further supported by the authors’ extensive previous research on diverse busbar configurations. While this study primarily validates the multi-platform software using the 5BB layout, the comparative context for other systems, such as 1BB, is detailed in our earlier work [22]. It was established that increasing the number of busbars is essential for mitigating ohmic losses, a principle that served as a fundamental basis for the predictive algorithms developed in the current tool.

## Figures and Tables

**Table 1:** Elemental composition of selected areas of the solar cell, namely: (1) surface and (2) busbar.

The Area	Weight Fraction of Elements, %	
	Si	Ag
1 (Figure 6a)	100	-
2 (Figure 6b)	-	100

**Table 2:** Electrical parameters of solar cells (where U_oc_—open-circuit voltage, I_sc_—short-circuit current, U_m_—voltage at maximum power), I_m_—current at maximum power, R_s_—series resistance, P_m_—maximum power, FF—fill factor, E_ff_—conversion efficiency).

Number of Solar Cell	Parameters							
	I_sc_ [mA]	U_oc_ [mV]	I_m_ [mA]	V_m_ [mV]	P_m_ [mW]	R_s_@U_oc_ [mΩ]	FF [-]	E_ff_ [%]
1	9710.3	662.2	8511.5	454.7	3869.9	14.5	0.602	16.00
2	9673.0	666.0	8554.5	465.3	3980.3	14.3	0.618	16.49
3	9699.9	666.1	8469.0	463.9	3928.7	14.5	0.608	16.29
4	9637.8	666.2	8459.8	475.7	4024.2	10.7	0.627	16.67
5	9651.1	664.9	8276.5	452.1	3742.0	16	0.583	15.48
6	9673.5	666.2	8343.2	445.0	3713.0	16.3	0.576	15.37
7	9590.3	667.0	8171.4	450.8	3683.6	16	0.576	15.26
8	9692.7	667.3	8183.4	450.4	3686.0	16	0.570	15.26
9	9611.5	667.1	8171.4	454.6	3714.6	15.8	0.579	15.42
10	9662.5	668.9	8196.9	458.3	3756.3	15.4	0.581	15.60
11	9383.5	673.0	8319.7	461.0	3835.4	15.2	0.607	16.07
12	9446.7	672.8	8320.8	460.2	3829.6	15.2	0.603	16.08
13	9447.7	676.5	8029.8	467.6	3754.8	15.2	0.587	15.83
14	9406.6	674.5	8082.5	456.2	3686.9	16.3	0.581	15.46
15	9431.3	671.2	8256.5	467.2	3857.3	14.7	0.609	16.23
16	9419.6	667.8	8149.0	473.2	3856.5	13.7	0.613	16.16
17	9558.7	669.8	8020.0	461.1	3698.4	14.9	0.578	15.48
18	9525.4	670.7	7763.2	495.8	3849.1	12	0.602	16.14
19	9618.8	670.6	8189.3	456.9	3741.3	16.1	0.580	15.61
20	9569.4	672.1	8223.0	440.9	3625.3	17.3	0.564	15.21

**Table 3:** Minimum and maximum values input parameters.

Input Parameter	Minimum Value	Maximum Value
Contact-emitter potential (U_ce_)	0 mV	160 mV
Width of the path (w)	0 mm	5 mm
Distance between the collecting paths (d)	0 mm	10 mm
Contact current density (J)	0 mA/cm^2^	30 mA/cm^2^

**Table 4:** Sample results obtained during system operation (chosen examples).

Sample No.	Input Values: w = 0.12 mm d = 1 mm J = 30 mA/cm^2^	Calculated Values		Measured Values		Errors			
						Absolute		Relative	
	U_ce_	R_c_	ρ	R_c_	ρ	R_c_	ρ	R_c_	ρ
1	22.0	158.4	13.3	158	13.2	0.4	0	0.25	0
	57.1	483.12	40.26	474	39.5	9.12	0.76	1.88	1.88
	77.1	555.12	46.26	555	46.3	0.12	0.04	0.02	0.08
	116.2	836.64	69.72	837	69.7	0.36	0.02	0.04	0.02
	149.4	1075.68	89.64	1076	89.6	0.32	0.04	0.03	0.05
2	23.1	166.32	13.86	160	13.4	6.32	0.46	3.79	3.32
	57.6	414.72	34.56	408	34.0	6.72	0.56	1.62	1.62
	71.3	513.36	44.4	521	44.4	7.64	1.62	1.48	3.77
	97.7	703.44	58.62	689	57.4	14.44	1.22	2.05	2.08
	124.1	893.52	74.46	872	72.7	21.52	1.79	2.40	2.36
3	27.3	196.56	16.38	197	16.4	0.44	0.02	0.22	0.12
	44.9	323.28	29.94	323	26.9	0.28	0.04	0.08	0.14
	87.4	629.28	52.44	642	52.4	12.72	1.76	2.02	3.35
	126.5	910.8	75.9	911	75.9	0.02	0.00	0.02	0.00
	134.4	967.68	80.64	953	79.4	14.68	1.24	1.52	1.54
4	16.1	115.92	9.66	116	9.7	0.08	0.04	0.07	0.42
	61.5	442.91	36.9	443	36.9	0.10	0.00	0.02	0.00
	92.3	64.56	55.38	657	54.8	7.56	0.58	1.14	1.05
	133.9	964.09	80.34	942	78.5	22.08	1.84	2.29	2.29
	150.6	1084.32	90.36	1044	88.2	40.32	2.16	3.72	2.39

## Data Availability

The original contributions presented in this study are included in the article. Further inquiries can be directed to the corresponding author.
